# Structural insight in the inhibition of adherence of F4 fimbriae producing enterotoxigenic *Escherichia coli* by llama single domain antibodies

**DOI:** 10.1186/s13567-015-0151-x

**Published:** 2015-02-24

**Authors:** Kristof Moonens, Imke Van den Broeck, Emmanuel Okello, Els Pardon, Maia De Kerpel, Han Remaut, Henri De Greve

**Affiliations:** Structural & Molecular Microbiology, Structural Biology Research Center, VIB Brussels, Belgium; Structural Biology Brussels, Vrije Universiteit Brussel, Pleinlaan 2, 1050 Brussels, Belgium; College of Veterinary Medicine, Animal Resources and Bio-security, Makerere University, P.O. Box 7062, Kampala, Uganda

## Abstract

Enterotoxigenic *Escherichia coli* that cause neonatal and post-weaning diarrhea in piglets express F4 fimbriae to mediate attachment towards host receptors. Recently we described how llama single domain antibodies (VHHs) fused to IgA, produced in *Arabidopsis thaliana* seeds and fed to piglets resulted in a progressive decline in shedding of F4 positive ETEC bacteria. Here we present the structures of these inhibiting VHHs in complex with the major adhesive subunit FaeG. A conserved surface, distant from the lactose binding pocket, is targeted by these VHHs, highlighting the possibility of targeting epitopes on single-domain adhesins that are non-involved in receptor binding.

## Introduction, methods and results

F4 fimbriae are expressed on the cell surface of enterotoxigenic *E. coli* (ETEC) to mediate attachment towards carbohydrate receptors localized on the enterocytes of piglets [1]. ETEC strains are responsible for significant death and morbidity in neonatal and post-weaned piglets by causing severe, watery diarrhea [2], resulting worldwide in severe economic losses in pig industry. Clinical symptoms are generated by the action of enterotoxins: the heat-labile enterotoxin (LT) and/or two unrelated heat-stable enterotoxins (STa and STb) [3]. F4 fimbriae are assembled by the conserved chaperone-usher pathway [4] and composed out of a major adhesive subunit FaeG, resulting in the exposure of many hundreds consecutive binding surfaces along the flexible F4 fimbrial structure. In the final quaternary structure fimbrial subunits complement the incomplete immunoglobulin-like fold of one another by donating an N-terminal donor strand *in trans* to the preceding subunit [4]. Three naturally occurring serological variants of F4 fimbriae (F4_ab_, F4_ac_ and F4_ad_) exist that differ in the primary sequence of FaeG, with each variant featuring a related but yet different binding and hemagglutination profile [5,6]. The FaeG structure was earlier determined and shows a conserved immunoglobulin-like fold, typical for fimbrial subunits, on which a subdomain comprising two short β-strands and two α-helices is grafted [7]. Most of the variability is localized on and around this additional subdomain and recently we elucidated the co-complex structure between FaeG_ad_ and lactose (Moonens *et al.*, under review), demonstrating the glycan ligand is interacting in a binding site located on the additional subdomain. A commercial vaccine containing F4 fimbriae is currently available on the market and provides maternal passive immunity against *E. coli* induced neonatal diarrhea [8]. To this date no commercial vaccine or prevention strategy against post-weaning diarrhea caused by F4 fimbriated ETEC is yet available. Within our research group anti-ETEC antibodies were generated by fusing four different variable domains of llama heavy chain-only antibodies (V1-4), raised against FaeG_ntd/dsc, ac_ and panned against all three FaeG_ntd/dsc_ variants (first variant ac, than ad and finally ab), to the Fc domain of a porcine immunoglobulin IgA. The resulting four VHH-IgA constructs were subsequently expressed in *Arabidopsis thaliana* seeds and fed to piglets [9]. The oral feed-based passive immunization strategy protected piglets as demonstrated by the progressive decline in shedding of F4 positive ETEC bacteria, the significantly lower immune responses of the piglets to F4 fimbriae which suggest a reduced exposure to the ETEC pathogen, and a significantly higher body weight in comparison with control piglets [9]. It was demonstrated as well that seed extracts containing VHH-IgA antibodies could inhibit the attachment of F4 positive ETEC strains to porcine gut villous enterocytes in vitro [9].

**Figure 1:**
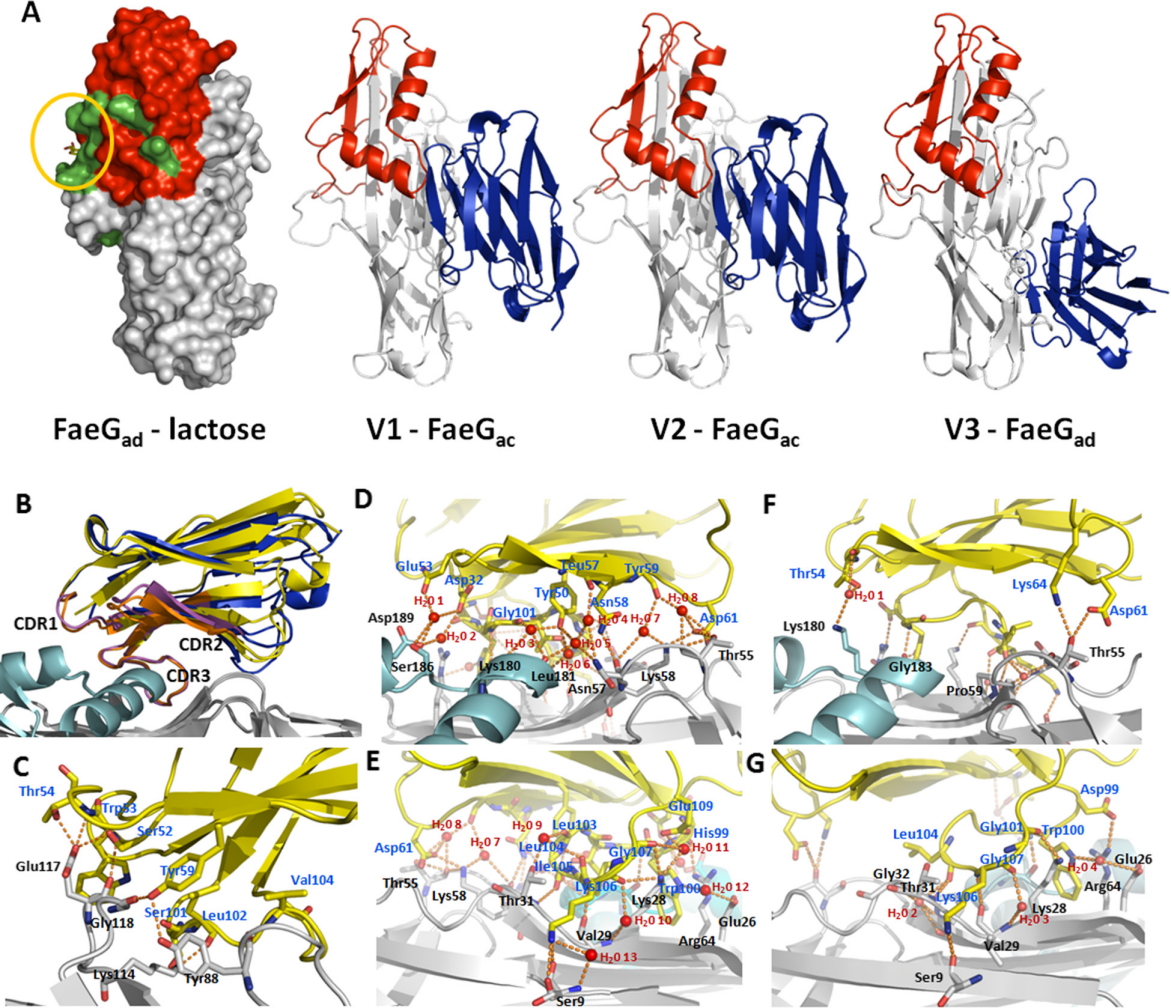
**Crystal structures of inhibitory VHHs in complex with the major adhesive subunit FaeG. (A)** From left to right, molecular surface representation of the structure of FaeG_ad_ in complex with lactose (stick representation), co-complex structure of V1 with FaeG_ac_, co-complex structure of V2 with FaeG_ac_ and finally the co-complex structure between V3 and FaeG_ad_. The orientation of FaeG in each panel is identical, the additional binding domain grafted onto the FaeG immunoglobulin-like core is colored red and the VHH in the last three panels is colored in blue. Variation between the different FaeG variants is colored on the molecular surface of FaeG_ad_ in green. The lactose binding site on the surface of FaeG_ad_ is indicated by a yellow circle for clarity. **(B)** Comparison of the binding conformation of V1 (yellow) and V2 (blue). The different CDR regions of the VHHs are indicated and colored in orange (V1) and purple (V2). In V2 the CDR2 is shifted upwards and located at a further distance from the FaeG surface, whereas the conformation of the CDR3 is near identically traced. **(C)** Close-up on the interactions formed in the complex between V3 and FaeG_ad_. **(D-G)** Interactions formed between V1 and FaeG_ac_ **(D,E)** and V2 and FaeG_ac_ **(F,G)** in different orientations. In each panel FaeG is colored gray and the additional variable subdomain in cyan. VHHs are depicted in yellow and water molecules are represented as red spheres. Interacting residues of the VHH and FaeG adhesin are labeled respectively blue and black and shown as stick model with nitrogen atoms colored blue and oxygen atoms in red. Hydrogen bonds are depicted as orange dashed lines.

Using X-ray crystallography we investigated the mechanism of action of the isolated VHHs that inhibit the F4 fimbriae-mediated binding. Stable self-complementing FaeG constructs of all three variants (FaeG_ab_, FaeG_ac_ and FaeG_ad_), in which the N-terminal donor strand was swapped to the C-terminus via a short tetrapeptide DNKQ linker, were expressed and purified as described earlier [10]. Complexes between the different purified VHHs [11] and the self-complementing variants of FaeG were produced by incubating them together with excess VHH and separating the complex by size exclusion chromatography. Crystals were obtained for the complexes of FaeG_ac_-V1, FaeG_ac_-V2 and FaeG_ad_-V3 in respectively condition A10 of the Clear Strategy Screen I HTS-96 (Molecular Dimensions), C4 of the JBScreen Basic HTS (Jena Bioscience) and E12 of the Morpheus Screen HT-96 (Molecular Dimensions) using the sitting drop damp diffusion method. Diffraction data were indexed using XDS [12] (Table 1) and further prepared and scaled using respectively Pointless and Scala [13]. The phase problem was solved with the molecular replacement method by Phaser [13] with the coordinates of the self-complementing FaeG_ad_ (PDB identifier 3HLR) and a llama single domain antibody as search models. The resulting models of the co-complexes were further improved by manually building in the molecular graphics program COOT [14] and refined using Refmac5.5 [13] (Table 1). All three inhibitory VHHs interact with conserved epitopes on the FaeG surface (Figure 1A). V1 and V2 interact with nearly similar epitopes constituted of residues of the conserved immunoglobulin-like core domain and conserved residues of the additional variable subdomains (Figure 1A). V3, in contrast, is only interacting with a patch of amino acids located on the Ig-like conserved core domain (Figure 1A). The heavy chain-only antibodies V3 and V4 only differ in one amino acid substitution (Lys 100 Arg), and analysis of the crystal structure of V3 in complex with FaeG_ac_ revealed Lys100 is not involved in any stabilizing interactions. When comparing the structures of the FaeG-VHH complexes with the recently determined FaeG_ad_-lactose structure (Moonens et al., under review) it is obvious that the binding of the different VHHs onto FaeG_ad_ does not target the lactose binding site and hence the F4_ad_ fimbriae are not obstructed in their carbohydrate binding capability (Figure 1A). All VHHs are targeting conserved patches on FaeG, and since the specificity of the different FaeG variants has been localized on the additional binding domain [15] we can conclude that the inhibitory mechanism of the VHHs is not governed by directly interfering with the carbohydrate binding site.

**Table 1:** **Crystal parameters and data processing statistics**

**Complex**	**FaeG**_**ac**_ **-V1**	**FaeG**_**ac**_ **-V2**	**FaeG**_**ad**_ **-V3**
Wavelength	0.98	0.98	0.98
Beamline	Soleil - Proxima 1	Soleil - Proxima 1	Diamond - IO3
Space group	P 3 2 1	P 3 2 1	P 2 2_1_ 2_1_
a, b, c (Å )	145.5, 145.5, 38.9	145.8, 145.8, 37.9	79.8, 95.2, 113
α, β, γ (°)	90, 90, 120	90, 90, 120	90, 90, 90
Resolution (Å)	47.62 – 1.55 (1.63 – 1.55)	47.74 - 1.89 (2.0 - 1.89)	29.54 – 2.61 (2.67 – 2.61)
R_meas_ (%)^a,b^	4.9 (56.9)	12.3 (198.4)	10.5 (117)
No. of unique reflections^b^	68481 (9923)	36784 (5212)	26553 (1559)
Average I/σI	24.8 (4.2)	14.3 (1.4)	16.1 (1.1)
CC (1/2)	100 (92.2)	99.9 (79.6)	99.8 (49)
Multiplicity^b^	11.2 (10.9)	20.2 (19.3)	10.6 (3.8)
Completeness (%)^b^	99.9 (99.0)	99.7 (97.7)	98.3 (79.6)
Wilson B-factor	24.9	35.6	63.4
R_work_/R_free_(%)^c, d^	16.9/19.3	18.9/22.7	20.5/24.5
Average B-factor (Å^2^)	20.2	28.3	35.7
R.m.s. deviations			
Bond lengths (Å)	0.026	0.021	0.012
Bond angles (°)	2.715	2.150	1.607
No. Atoms (except H)			
Protein	2778	2752	5293
Water	305	105	29
Residues in allowed regions	100	98.6	98.7
(%) of Ramachandran plot			
PDB entry	4WEM	4WEN	4WEU

^a^R_*meas*_ = Σh (*n*h/*n*h-1) Σl |*Ihl* - < *Ih* > |/ Σh Σl < *Ih*>, where *n*h = the number of observations for reflection **h**,Ihl = the intensity for observation **l** of reflection **h**, and < Ih > = the average intensity for reflection **h**.

^b^Statistics for outer resolution shell are given in parenthesis.

^c^R_work_ = Σhkl ||Fobs | - |Fcalc|| / Σhkl |Fobs|.

^d^R_free_ is defined as above but calculated for 5% of randomly chosen reflections that were excluded from the refinement.

**Figure 2:**
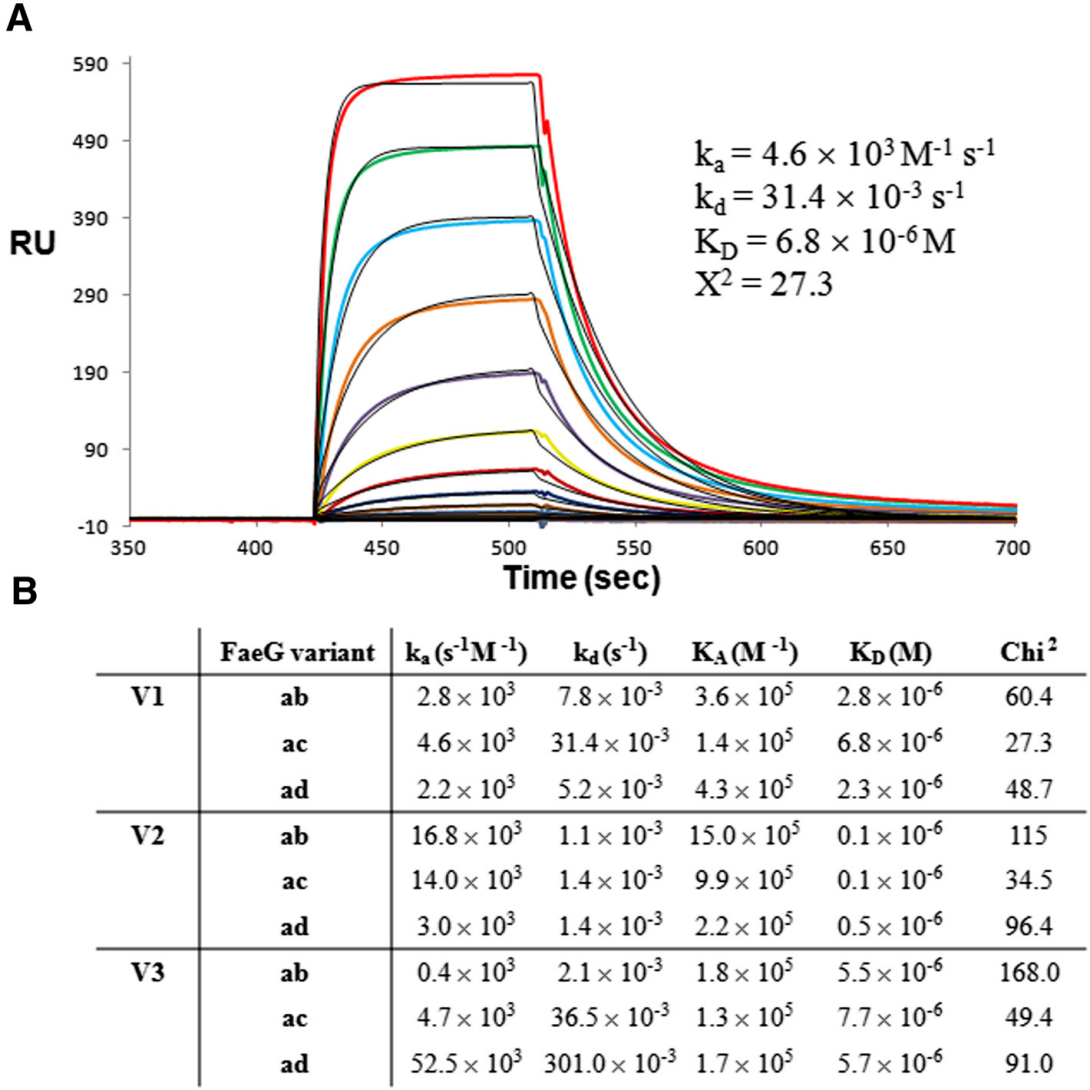
**Interaction between inhibitory VHHs and the different FaeG variants. (A)** Typical set of sensorgrams obtained when assaying the interaction between inhibitory VHHs and FaeG (here depicted is the interaction between V1 and FaeG variant ac). Sensorgrams were obtained by injecting varying concentrations of FaeG (50 μM to 1.5 nM) over covalently immobilized VHHs. The curves were fitted using a 1:1 Langmuir binding model. Fitted curves are shown in black, while the original data is represented by the colored curves. **(B)** Overview on the association (k_a_) and dissociation (k_d_) rate constants, and association (K_A_)/dissociation (K_D_) constants of the interaction between the different VHHs and FaeG variants.

The strength of the interaction between the different VHHs and FaeG variants was determined using surface plasmon resonance (Figure 2). The surface of a CM5 sensor chip (GE Health Care) was activated, the different VHHs immobilized on flow channel 2 via primary amine groups and finally residual unreacted active ester groups were deactivated, all according to manufacturer’s protocol. As a control, flow channel 1 was as well activated and deactivated. The different FaeG variants were flowed over the chip surface in a two-fold dilution series in HBS buffer (10 mM HEPES, 150 mM NaCl, 1 mM EDTA, 0.005% Tween20, pH 7.4) at a flow rate of 10 μL/min at 25°C. The obtained sensorgrams of the subtracted (Fc2 - Fc1) signals were fitted using a Langmuir binding isotherm with a 1:1 stoichiometry, from which the kinetic rate constants k_a_ and k_d_ were obtained (BIAeval software; Biacore AB). Affinities varied from low μM for V1 and V3, to high nM for V2 (Figure 2B). These experimentally determined affinities differ significantly from the low nM dissociation constants by which VHHs typically recognize their target antigens [16,17]. This discrepancy may arise because of the VHH selection procedure. The earlier described anti-FaeG VHHs were selected consecutively against all three FaeG variants and instead of selecting binders that demonstrated the highest affinity towards only one FaeG variant, most likely VHHs with moderate affinity against all three variants were obtained during the selection procedure. Even amongst the conserved surfaces of the different FaeG variants small structural perturbations are observed. A panning procedure selecting binders against all FaeG variants would select VHHs interacting with an averaged FaeG structure, but not necessarily with high affinity.

The detailed interactions between the inhibitory VHHs and the major adhesive subunit FaeG of F4 fimbriae are shown in Figure 1B-G. The interaction between V3 and FaeG_ad_ is mediated mainly by direct hydrogen bonds between the amino acid stretches Ser52-Thr54, Tyr59 and Ser101-Val104 of V3, that correspond with respectively the complementarity determining regions CDR2 and CDR3, with the amino acid residues Tyr88, Lys114 and Glu117-Gly118 from FaeG_ad_ (Figure 1C). Since V1 and V2 are targeting overlapping epitopes, we superimposed the two structures of V1-FaeG_ac_ and V2-FaeG_ac_. The two VHHs are directed in an identical orientation to interact with the FaeG adhesin (Figure 1A). Only the conformation of the CDR2 is significantly altered and is more distant from the FaeG molecular surface in the V2-FaeG_ac_ co-complex structure (Figure 1B). In the V1-FaeG_ac_ structure a large amount of water molecules, eight in total, are observed in the interaction interface formed by the CDR2 region and those are all involved in inter molecular hydrogen bond formation between V1 and FaeG_ac_ (Figure 1D). In addition, only four direct hydrogen bonds are formed between the FaeG_ac_ and CDR2 of V1 (Figure 1D). In contrast, the V2-FaeG_ac_ co-complex structure in the same interface exhibits only one water molecule and three direct hydrogen bond interactions (Figure 1F). As there is no sequence conservation between the CDR2s of V1 (SEG**G**ILN) and V2 (TNT**G**VTE) this difference in binding mode is not so surprising. The VHH-FaeG interaction surface on the other side is bordered by the CDR3 region that is nearly conserved amongst V1 (**AA**SH**WGTLLIKGI**E**H**) and V2 (**AA**TD**WGTLLIKGI**D**H**). Again more water molecules are observed in the interaction interface between V1-FaeG_ac_ compared to V2-FaeG_ac_ (5 versus 3 water molecules); however more direct interactions (6 direct hydrogen bonds) are formed in both complexes (Figures 1E and G). Unexpectedly although in the V1-FaeG_ac_ complex many more direct and indirect interactions are formed the affinity between V1 and FaeG_ac_ is 70 times lower compared to the affinity of V2 for FaeG_ac_ (Figure 2B). The inclusion of more water molecules in the V1-FaeG_ac_ complex binding interface might have an unfavorable effect on the interaction, thereby reducing affinity.

## Discussion

**Figure 3:**
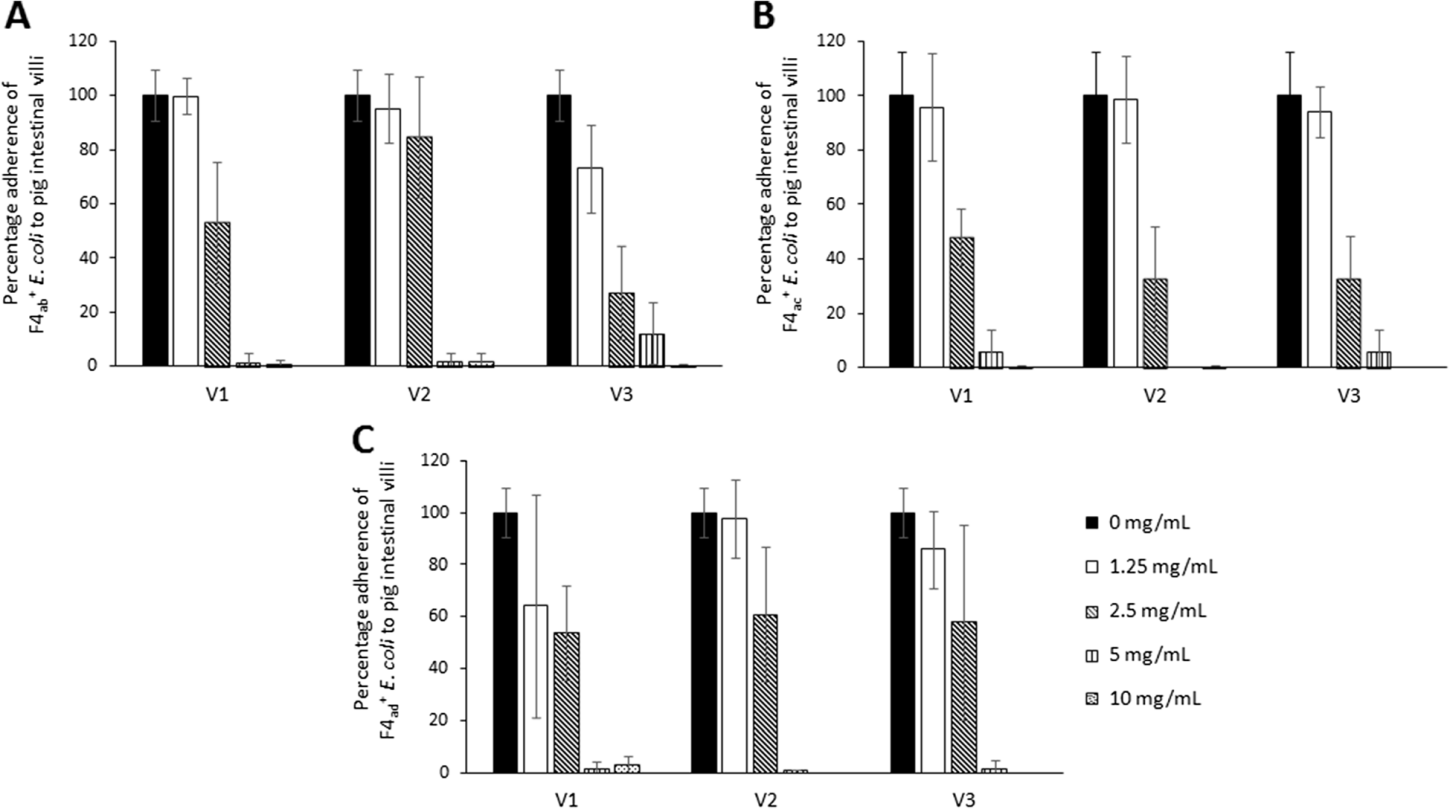
**VHH inhibition of the adherence of F4 fimbriated** ***E. coli*** **to piglet enterocytes** ***in vitro.*** Quantitative analysis of bacterial adherence to piglet enterocytes of strains expressing F4_ab_ **(A)**, F4_ac_ **(B)** and F4_ad_ **(C)** fimbriae. A two-fold dilution series of monomeric VHHs was added to 4 × 10^8^ F4^+^ cells and incubated during one hour with an average of 50 villi. Villi were examined by phase-contrast microscopy at a magnification of 600× and the number of bacterial cells adhering to 250 μm villi length (5 repeated reads for each test sample) were counted and plotted as a percentage of wild type binding.

Crosslinking of bacteria by antibodies, like for example in *Vibrio cholera* [18] and *Streptococcus mutans* that causes dental carries [19], has been shown to be important for mediating protection. In contrast the neutralizing activity of polyclonal IgM in the attachment of influenza virus to target cells is due to steric hindrance [20]. When the four VHHs were covalently coated on magnetic beads, specific agglutination with the three variants of F4^+^ bacteria was observed [9]. In a hemagglutination based assay purified monovalent VHH were unable to inhibit the interaction of guinea red blood cells and F4 fimbriated bacteria (results not shown). However, in an in vitro adherence assay the VHHs were able to prevent the attachment of F4 fimbriated bacteria to piglet enterocytes (Figure 3). The inhibition of adherence by VHHs was fully reversible by the addition of monomeric FaeG, demonstrating the specificity of the VHHs. Concentrations of VHH were identical in both experiments and these results indicate the VHHs are able to abrogate binding towards enterocyte based receptors but cannot prevent the cross-linking of red blood cells by F4 fimbriated bacteria. With crystallographic evidence we demonstrated the different VHHs contact conserved patches on the FaeG surface that are non-involved in carbohydrate binding. We conclude the mechanism of inhibition is mostly attributed to steric hindrance of the interaction between F4 fimbriae and enterocyte based receptors and to a lesser extent cross linking of F4 expressing bacterial cells.

VHHs possess a range of advantages compared to more commonly employed prophylactic treatments. They recognize their target antigen often with high affinity, whereas organic compounds often require several intensive rounds of structure-based chemical optimization to attain a reasonable binding affinity. However in this study the measured affinity of anti-F4 VHHs was relatively weak, probably due to the VHH panning procedure that selected out reasonable binders against all three FaeG variants. The binding site interface contains many water molecules, a peculiar binding mode that potentially could facilitate recognition and interaction with all three FaeG variants but at the cost of affinity. Since the receptor binding subdomain is highly variable, during the selection rounds VHHs interacting at a conserved surface will be selected since they recognize all three variants. However more often anti-adhesives target the carbohydrate binding site to efficiently prevent the interaction between the bacterial cell and host receptors [21]. In future this understanding will help to further enhance the prophylaxis treatment against F4 fimbriated ETEC by selecting specific VHHs with increased affinity and inhibitory capacity by selecting them against the carbohydrate binding site and against solely one FaeG variant at a time. Recently we could completely inhibit the in vitro attachment of F18 fimbriae positive *E. coli* to piglet enterocytes by raising VHHs against the carbohydrate binding site of the F18 fimbrial adhesin FedF [22]. Krüger et al. demonstrated single-chain Fv (scFv) antibody fragment expressing lactobacilli could markedly reduce the *Streptococcus mutans* bacteria counts and caries scores in a rat model [23]. Likewise, expression of VHHs on lactobacilli can provide an alternative approach for in vivo passive immunity against F4 fimbriated ETEC. Nevertheless, although the selected VHHs (V1, V2, V3 and V4) against the three F4 variants do not exhibit a high affinity and target mainly the conserved immunoglobulin-like core domain of the FaeG subunit variants, they are protecting weaned piglets against infection by F4 positive ETEC strains regardless of the F4 variant that is expressed by the ETEC strain.
